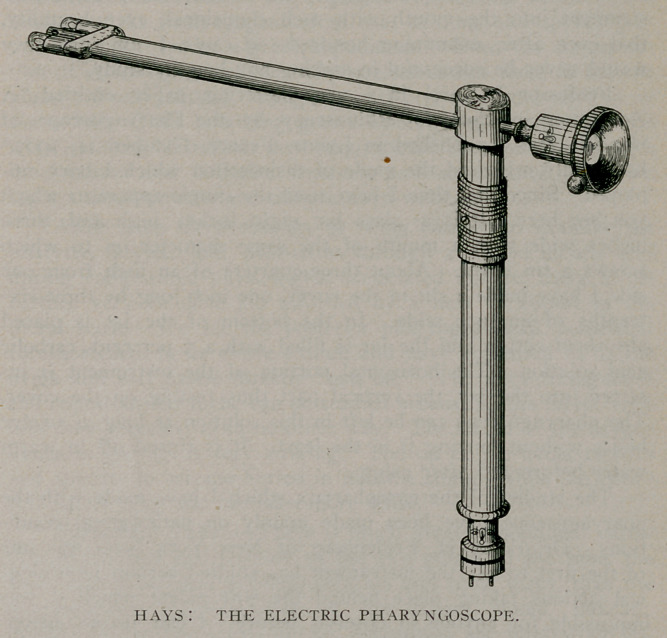# Studies of the Nasopharynx with a Brief Description of the Electric Pharyngoscope1Read before the Hospital Medical Society, Rochester, N. Y., December 23, 1909.

**Published:** 1910-02

**Authors:** Harold Hays

**Affiliations:** New York


### BUFFALO MEDICAL JOURNAL.

VOL. LXV. FEBRUARY, 1910. NO. 7

### ORIGINAL COMMUNICATIONS.

### Studies of the Nasopharynx with a Brief Description of the Electric Pharyngoscope[1]

BY HAROLD HAYS, A.M., M.D., New York

THE examination of the nasopharynx by means of the rhinoscopic mirror, has not allowed us to observe very closely many of the physiological actions or the pathological alterations in this region. To the expert, such an examination is far from impossible, but numerous difficulties arise which limit the time and extent of observation, such as a narrow nasopharyngeal space, the smallness of the mirror, the film of condensation that collects on the mirror, the manipulation necessary to get a view of the most important parts, and so on.

Because of these difficulties, particularly because of the unreliability of the usual observations I had attempted to make on the eustachian tubes, I sought to devise some instrument which would admit of a closer view of the parts to be examined with a minimum of discomfort to the observer and the patient. The instrument which I finally worked out is the one before me which I have called a pharyngoscope, as it was primarily intended for the examination of the vault of the pharynx and the surrounding parts. However, the uses of the instrument are not by any means confined to these parts, for most beautiful and defined views of the larynx can also be seen without the uncomfortable procedures usually necessary for such an examination.[2]

I shall give but a brief description of the instrument here, quoting from my paper on Pharyngoscopy, which appeared in the *New York Medical Journal,* August 21, 1909, reserving the major explanation of its manipulation until I have demonstrated it upon the patients here tonight.

The latest model of the pharyngoscope is composed of a horizontal and a vertical shaft which join each other at right angles, at the outer third, so that the instrument may be used as

1. Read before the Hospital Medical Society, Rochester, N. Y., December 23, 1909.
2. The *American Journal of Surgery,* May, 1909; the *Laryngoscope,* July, 1909.

a tongue depressor. The inner portion of the horizontal shaft consists of a central circular tube with an electric light carrier on either side, the three components being incorporated in a flat piece of metal. From the inner end project the two electric lights which are water tight, give an intense illumination and become only warm enough to keep vapor off the lens. In the central tube is inserted the telescope, which is made on the principle of the Otis cystoscope. To the eyepiece is attached a little metal ball which indicates the position of the lens. This horizontal shaft, including the telescope, is about eight inches long. The widest portion of the instrument, which is at the extreme inner end, is less than five-eighths of an inch, and the flat metal shaft itself measures less than one-half an inch in diameter. The vertical portion is about six inches long and half an inch wide. It is attached to the horizontal portion by a screw joint, and contains the wires for connection with the rheostat or dry cells. Near its upper portion is an arrangement for cutting off the electric current so that the lamps need not be turned on until the instrument is in the mouth.

On account of the cement which holds the lens in place, the instrument cannot be boiled, but, like the cystoscope, it is best disinfected by formalin. For that purpose a metal box is supplied with the instrument which is large enough to contain the horizontal shaft (except the eyepiece), which has a receptacle at one end for formalin tablets and cotton. These formalin tablets may be obtained from Schering & Glatz, New York City. For ordinary purposes the instrument is cleaned with lysol, 5 per cent. carbolic acid and alcohol.

The instrument is inserted into the mouth like a tongue depressor until the inner end of the telescope is about one-sixteenth of an inch from the pharyngeal wall. When once in place it is held firmly by the examiner and the patient is told to close his mouth and breathe quietly through his nose. As soon as the mouth is closed, it is observed that the muscles are relaxed and that the nasopharyngeal space is much enlarged. An excellent view of the parts to be seen can be obtained by gazing through the eyepiece of the instrument. In order to keep the patient from breathing in the examiner's face a small mica plate can be placed between the telescope and the horizontal shaft.

Since this last paper appeared on the subject I have been informed that some of the men have found difficulty in manipulating the instrument. After examining some 2,000 cases with no trouble at all, I must naturally feel that the fault is inherent in the man who is using the instrument and not in the instrument itself. There are two or three points of great importance:

1. The illumination must be as great as the lamps can stand.

2. The lights should not be turned on until the instrument is in the mouth.

3. The lamps should not touch any part of the throat.

4. Every manipulation should be made gently but firmly.

Many men have not been able to get sufficient illumination because they have used a 16-candle power lamp instead of a 32-candle power lamp on the rheostat. The former cannot be

HAYS: THE ELECTRIC PHARYNGOSCOPE.

turned down enough to give sufficient light. Of course, where dry cells or a wall plate is used, no trouble of this kind occurs.

The lights should not be turned on until the instrument is in the mouth. Being careful about this point makes a great difference. The throat reflexes are very delicate and therefore the nervousness of the patient is greatly exaggerated if he sees two burning lamps going into the throat, no matter how much assurance one gives him that they do not get hot. On the contrary, if the lamps are not lighted until out of the line of the patient's vision he imagines that only an ordinary tongue depressor is being used.

The third point is of equal importance for a sensation of warmth in the throat is liable to be exaggerated. If the patient should persist in changing his position so that the instrument cannot be held steadily in the mouth, it is better to remove it and try the procedure again.

The success which one attains in using such an instrument as the pharyngoscope depends greatly on the gentleness and the dexterity of his manipulations. I have seen men poke the instrument into the mouth with such clumsiness, even brutality, that even after examining hundreds of cases, I am sure they would never be successful in making any lengthy study.

Professor J. Garel in a very flattering paper entitled "A Revolution in Posterior Rhinoscopy by the Pharyngoscope of Harold Hays," published in Lyons, France, October 24, 1909,[1] has mildly criticised the mode of disinfection which I have employed. Since that time I have used the simple apparatus which you see here. It is a glass jar eight inches high and three inches wide with a mouth of the same diameter on to which screws a tin cover. About three-quarters of an inch from one side I have made a slit in the cover, one inch long by three-sixteenths of an inch wide. In the bottom of the jar is placed absorbent cotton and the jar is filled with a 5 per cent. carbolic acid solution. The horizontal portion of the instrument is inserted into the jar, the vertical part thus resting on the cover. The pharyngoscope can be left in this solution as long as twelve hours without hurting it in the least. It is rinsed off in warm water before and after using.

The studies of the nasopharynx which I have made with the pharyngoscope have been made mainly on pathological conditions. Dr. Percy H. Fridenberg of New York, who was one of the first to use the instrument has studied certain physiological actions taking place behind the soft palate which it was impossible for anyone to observe before. I can do no better than to quote a portion of his paper.[2]

"In fact, with superficial cocainisation of the uvula and pharyngeal wall, we can allow the instrument to remain in situ during a prolonged examination of the parts. After a few seconds, the subject seems hardly aware of its presence. I consider this a valuable feature and one which will be of great service in the examination of the pharynx and larynx during such physiological acts as yawning, deglutition, or pathological movement complexes, such as gag-

1. Une Revolution dans la Rhinoscopie Postérieure. Par le Pharyngoscope de Harold Hays. Par J. Gorel, Lyon Médical, October 24, 1909.

2. Pharyngoscopic Studies. Percy Fridenberg. *The Laryngoscope*, July, 1909.

ing, coughing, and so on. Some of these cannot possibly be studied by the usual indirect method of mirror laryngoscopy, as the mouth has to be held open and the mirror almost invariably interferes mechanically with some movements of the base of the tongue. Even where there is no actual pressure or even contact, the attention of the patient is concentrated on the hypothetical or expected interference with the automatic and natural coördination and sequence of muscular action. In a mechanism as finely organised as that of tone and speech production this must be a great impediment. Even the concentration of attention on the normal processes causes them to become unnatural and labored, losing the rapidity, ease, and rhythm of the reflex. Thus breathing grows labored and changes its rate when we try to count our own respirations. Locomotion changes its character and becomes rhythmic and accentuated when we study the movements and sensations in walking.

"Aside from the advantage of being enabled to examine the pharynx, larynx, and post-nares through a closed mouth and during complicated physiological associated motion, there is the further very valuable aid afforded by a new viewpoint in the literal sense. We see the posterior pharyngeal wall, somewhat foreshortened, in its entire extent, and have presented to us the uvula and soft palate directly "end on." The plica triangularis and supratonsillar niche can be studied because of the direct view into their recesses, as in no other way. The action of the azygos uvulæ in gaging, is very striking. The tip of the uvula recedes and appears to become buried in its own stump, while the entire soft palate rises and becomes more tense."

"*Eustachian Tubes.*—There is much greater individual variation in the size and position of the two pillars of the tube, the folds known as plica salpingo-palatina and tubal torus, than the diagrammatic representations of the atlases would indicate. Studies of the post-nares during deglutition are interesting, especially when there are adenoid masses in the epipharynx. Even slight gaging causes a marked reduction of the postnasal space by an approximation of the walls in parallelogram. The uvula and soft palate rise and straighten, there is some downward bulging of the upper part of the posterior pharyngeal wall and an advance toward the middle line of the entire tubal eminence and tori. As this becomes more pronounced, the opposing surfaces come into actual contact and one can clearly see how large masses of mucus are pressed out into the pharynx. Heretofore such studies were made only on the cadaver, or, in rare cases of extreme destruction of the turbinate bodies, by anterior rhinoscopy."

I shall classify the observations on the pathological conditions under three headings. The cases were studied in the New York Eye and Ear Infirmary, at the University and Bellevue Hospital Clinic and a great many in private practice where I use the pharyngoscope on almost every patient who comes for treatment.

The pathological conditions are: (1) abnormal conditions of the mucosa of the nasopharynx; (2) adenoid vegetations; (3) pathological conditions around the eustachian tubes and in the fossa of Rosenmüller.

1. *Abnormal Conditions of the Mucosa.*—The mucous membrane was atrophied or hypertrophied or there was a condition known as glandular hypertrophy. In the atrophic cases, mucus could be seen hanging in stringy masses either in the vault or extending from one side to the other. It presented a glary, sticky appearance. When this was washed away by postnasal douching and the pharyngoscope reinserted, one was immediately struck by the "roominess" of the entire postnasal space. The membranes looked drawn, they had lost their normal red appearance and oftentimes the hard and dry appearance could be traced to the turbinates and downward to the larynx. In cases of hypertrophy of the mucosa, considerable sensitiveness to any manipulation was often met with. The views that were obtained were in exact contrast to those above. The mucosa was unusually engorged and thickened and extremely moist. The thickening extended into Rosenmüller's fossa and frequently closed off the eustachian tubes. In one or two instances the boggy condition of the mucous membranes over the tubes was made manifest by passing an applicator through the nose and lifting up the membrane on it. It had fallen over the tubal opening in much the same manner as a reduplication of fat will take place on a double chin.

2. *Adenoid Vegetations.*—As the pharyngeal vault is so plainly seen, the diagnosis of adenoids becomes a very simple matter. The growth, as a rule, hangs down as a dependable mass from the pharyngeal vault. Its limits vary. But in children and in many adults the growth is limited anteriorly by the septum over which it hangs to a varying degree, sometimes giving complete occlusion to the nares, at other times occluding all but the inferior meatus. Posteriorly there are no definable limits, but in no instance have I seen an adenoid extend lower posteriorly than anteriorly. In other words, the greatest hypertrophy is in front and the growth merges with the pharyngeal wall posteriorly. The external limits of the adenoids are proportionate to the width of the nasopharynx and almost always some of the tissue extends into the fossæ of Rosenmüller and overhangs the eustachian tubes.

The adenoid is enveloped in a capsule, except where it is attached to the pharyngeal wall, and is composed of three main lobes and numerous smaller lobules. With the pharyngoscope in a sufficiently wide nasopharynx, the exact contour of the growth can be made out and the glistening capsule, entering into the crypts, can be plainly seen. The secretion of mucus from this surface goes on constantly.

The soft consistency of the adenoid, its location and its glandular structure, predispose it to constant irritation. The result is that even where hypertrophy does not take place, congestion and infiltration of the glandular tissue will allow it to increase in size to such an extent that there are intermittent periods of complete and partial nasal obstruction. A large adenoid growth need not obstruct all the time, but is sure to obstruct some of the time.

It is a common custom, unfortunately, among a great many men, to make a diagnosis of adenoid hypertrophy or obstruction, merely because the patient has always been a mouth-breather, particularly when the patient has a narrow nasopharynx in which a diagnosis with the mirror is nil. Many times, more especially in adults, digital examination but augments a wrong conclusion based on a preconceived opinion. For example, a short time ago a young man of nineteen was referred to me for the removal of his adenoids. I have no hesitation in saying that if I had not had the pharyngoscope I should have said positively that he had adenoids. He had the blank staring expression so often seen, the nose was narrow and he breathed with the mouth open. Moreover, he was in the habit of dribbling mucus on his pillow at night and suffered from so-called catarrh. Examination of the nasopharynx with the pharyngoscope showed a vault perfectly free from adenoid tissue. On further examination it was seen that he had a deformed dental arch with an extreme concavity of the hard palate. This, together with a narrow nose, was sufficient to account for the mouth-breathing. Since I saw this patient two other cases have come to my notice with somewhat similar history in which no adenoids were found.

A small adenoid growth, situated over the upper portion of the septum, about the size of a hazelnut, is very commonly overlooked. The growth is never large enough to cause obstruction to respiration and seldom gives any symptoms aside from a constant secretion of mucus. The rhinoscopic mirror may at times reveal the presence of the growth, but, as I have said, the diagnosis is often not made and the patient is treated for various intranasal conditions which do not relieve the symptoms. Often, on inquiry, one is told that the adenoids were operated upon. Therefore many times this little mass of tissue is a remnant of an hypertrophied piece of adenoid tissue which had not been removed. I have seen this state of affairs very frequently, the picture with the pharyngoscope being so clear that there is no possibility of a mistaken diagnosis. The simple procedure of removing this piece of tissue will frequently alleviate the symptoms without resorting to other operative measures.[1]

Among the commonest causes of tubal obstruction is adenoid or lymphoid hypertrophy in the fossa of Rosenmüller, on the eustachian eminence or in the mouth of the tube. Even where the pharyngeal vault is perfectly clear, a small adenoid mass situated in close proximity to the tube will cause pathological changes in the tube itself, resulting in the persistence of a middle-ear suppuration or resulting in a middle-ear catarrh with tinnitus and subsequent deafness. Once the offending mass is removed the ear changes retrogress and a normal, or nearly normal condition, results. An example of such a change is illustrated in the case of a young woman of twenty-eight who suffered from diminution of hearing and deafness in the left ear for one year. On examination with the pharyngoscope a small adenoid, springing from the left eustachian eminence and overhanging the tube, was found. Removal of this mass with the curet resulted in the establishment of normal hearing and subsidence of the tinnitus.

3. *Pathological Conditions Around the Eustachian Tubes and in the Fossa of Rosenmüller.*—By far the most interesting studies I have made have been those of the eustachian tubes and fossæ of Rosenmüller, particularly in relation to chronic catarrhal otitis media. The usual observations have been practically useless as a basis of treatment, for not only was that part of the naso-pharynx incompletely studied but applications to these regions were largely a matter of guess work. Almost all my private patients have their tubes catheterised under direct vision (at least the first time), by placing the pharyngoscope in the mouth and passing the catheter through the nose and many of the applications of medicaments are made in the same manner, so that no matter whether the medicine be caustic or soothing agent, the right spot is reached. For example, in one case where there was a prolapsus mucosæ of the tubal eminence, I was able to pass a fine applicator dipped in trichloracetic acid through the inferior meatus and applied it to the drooping mucous mem-

---

1. The Diagnosis of Adenoids in Children and in Adults. Harold Hays, *Journal of Ophthalmology, Otology and Laryngology*, August, 1909.

brane. The definite white streak which followed told its own story.

The eustachian tubes, eminences and fossa posterior to it present all of the gradations of inflammation and pathological alteration which are present simultaneously in other parts of the nasopharynx. The altered mucosa may be in a more or less advanced state than the surrounding parts and any advanced change in this location is bound to bring about some degree of abnormality in the middle-ear. I have seen many cases in which there was no ascertainable abnormality in the nose or throat, other than around the tubes, in patients with chronic middle-ear catarrh, where treatment directly to the tube brought about a cure of the ear condition where no other treatment, such as intratympanic massage, was attempted.

The pathological conditions in and around the tube which were plainly seen and studied with the pharyngoscope include congestion, anemia, hypertrophy, lymphoid hypertrophy, atrophy, adenoids, stenosis of the tube, tumor of the nasopharynx (probably specific) occluding the left tube, bulging posterior pillar of the fauces, due to hypertrophied tonsils and causing obstruction; ulceration on the posterior pillar with direct extension of pus into the tube and causing an acute otitis media and, finally, a common but often unrecognised condition—the presence of adhesions in the fossa of Rosenmüller.

The various pathological conditions mentioned above need little comment for it is more a question of seeing them and studying them, than being able to describe the exact pathology of conditions which are similar to those in mucous membranes elsewhere. The chief point I wish to bring out is that I have been able to make exact pathological diagnoses by means of the pharyngoscope, which otherwise would have remained in the obscurity which has characterised the majority of the abnormal conditions of the nasopharynx.

11 West Ninety-First Street.

## Figures and Tables

**Figure f1:**